# Oxalate in Foods: Extraction Conditions, Analytical Methods, Occurrence, and Health Implications

**DOI:** 10.3390/foods12173201

**Published:** 2023-08-25

**Authors:** Neuza Salgado, Mafalda Alexandra Silva, Maria Eduardo Figueira, Helena S. Costa, Tânia Gonçalves Albuquerque

**Affiliations:** 1 Research and Development Unit, Department of Food and Nutrition, National Institute of Health Dr. Ricardo Jorge, Avenida Padre Cruz, 1649-016 Lisbon, Portugaltania.albuquerque@insa.min-saude.pt (T.G.A.); 2 Faculty of Pharmacy, University of Lisbon, Avenida Professor Gama Pinto, 1649-003 Lisbon, Portugal; 3 REQUIMTE-LAQV/Faculty of Pharmacy, University of Porto, Rua Jorge Viterbo Ferreira, 228, 4050-313 Porto, Portugal; 4 Research Institute for Medicines and Pharmaceutical Sciences (iMed.UL), Faculty of Pharmacy, University of Lisbon, Avenida Professor Gama Pinto, 1649-003 Lisbon, Portugal

**Keywords:** oxalate, foods, extraction conditions, analytical methods, occurrence, health implications

## Abstract

Oxalate is an antinutrient present in a wide range of foods, with plant products, especially green leafy vegetables, being the main sources of dietary oxalates. This compound has been largely associated with hyperoxaluria, kidney stone formation, and, in more severe cases, systematic oxalosis. Due to its impact on human health, it is extremely important to control the amount of oxalate present in foods, particularly for patients with kidney stone issues. In this review, a summary and discussion of the current knowledge on oxalate analysis, its extraction conditions, specific features of analytical methods, reported occurrence in foods, and its health implications are presented. In addition, a brief conclusion and further perspectives on whether high-oxalate foods are truly problematic and can be seen as health threats are shown.

## 1. Introduction

Oxalate is a chemical compound that can form soluble and insoluble salts in water. This substance is present in a wide range of foods, with plant products being the main sources of dietary oxalates [1]. In plants, it plays a relevant role in various functions such as calcium homeostasis; pH regulation; plant growth, development and protection; photosynthesis; and detoxification of heavy metals [2,3].

According to the literature, various methods have been employed for the determination of oxalate in foods, including enzymatic assays [4,5,6,7,8,9,10,11], spectrofluorimetry [12], spectrophotometry, amperometry [9,13,14], electrochemical [15,16], capillary electrophoresis [17,18], titration [19,20,21,22,23,24,25,26,27], gas chromatography (GC) [28], and high-performance liquid chromatography (HPLC). HPLC is the most recently referenced method used for the determination of oxalates because of its high sensitivity, accuracy, versatility, and reliability, despite being expensive to purchase, repair, and maintain [29]. Conversely, despite its lower sensitivity, spectrophotometry is an inexpensive, rapid, and simpler method, requiring only one main instrument [30,31]. Accurate measurement of oxalate in foods is extremely dependent on its extraction, the first step in oxalate analysis [1]. Despite being two completely different methods, HPLC and spectrophotometry extraction conditions of total and soluble oxalates in foods are similar. Regarding analytical conditions, they have different and specific features since they are completely distinct procedures.

Concerning oxalate occurrence in foods, green leafy vegetables are particularly relevant, and some are considered high-oxalate foods. For example, published oxalate values are 329.6–2350 mg total oxalates/100 g fresh weight (FW) for spinach [32,33,34,35,36,37,38,39], 1235 mg total oxalates/100 g FW for rhubarb [1], 874 [40] and 1458.1 [1] mg total oxalates/100 g FW for swiss chard, 1079 mg soluble oxalates/100 g FW for sorrel [41], and 300.2–721.9 mg total oxalates/100 g FW for taro leaves [34].

Considering human health, oxalates have been a concern for a long time due to their antinutritive effects and potential nephrotoxicity [42,43]. As antinutrients, oxalates restrict the bioavailability of some nutrients since they can bind to minerals, reducing their absorption and use [3,44]. Potentially toxic soluble oxalates are delivered to the kidneys and can form calcium oxalate crystals there, which can lead to hyperoxaluria and kidney stone formation, also known as nephrolithiasis or urolithiasis [3,45,46,47,48]. In more serious cases, systemic oxalosis has been reported, a phenomenon in which calcium oxalate crystals deposit in various organs, tissues, and bones, when renal function declines and excess oxalate exists in the bloodstream [49,50].

This review has the purpose of gathering a considerable amount of information about oxalate, focusing on its extraction and analytical conditions and content in various foods measured by HPLC and spectrophotometry. Optimization of these parameters for oxalate determination in foods is very relevant to achieve reliable and accurate results considering the impact that this antinutrient could have on human health, especially on patients with kidney stone problems.

## 2. Oxalates

Oxalate, or oxalic acid, is an antinutrient present, commonly in trace amounts, in fruits, nuts, cereals, fungi, vegetables, aromatic plants, and beverages, with plant-based products being the main sources of dietary oxalates [1]. However, some plants have high quantities of these compounds. In this matter, green leafy vegetables, such as spinach, Swiss chard, and rhubarb, are highlighted [3,32].

Oxalate can form soluble and insoluble salts in water. When binding with sodium, potassium, and ammonium ions, it forms soluble oxalates, whereas with calcium, iron, and magnesium it precipitates, forming insoluble compounds and making these minerals unavailable for absorption. Despite this fact, for example, zinc absorption and metabolism do not appear to be affected. In general, insoluble salts in water can be freely dissolved in acid [44,51,52]. Regarding health, one of the most important insoluble salts is calcium oxalate, having two hydration forms, monohydrates and dihydrates, which impact the shape of its crystals [1].

**Figure 1:**
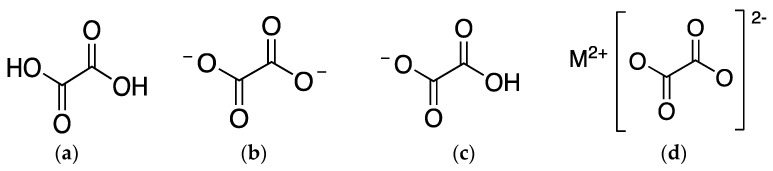
Chemical structure of (**a**) oxalic acid; (**b**) oxalate ion; (**c**) acid oxalate ion; and (**d**) oxalate salt, being M^2+^ a metallic cation.

Depending on the pH of the cell sap, the liquid inside the vacuole of plants where oxalates are mostly found, they can present different chemical structures (Figure 1). On the one hand, when pH is 2, acid oxalate is the main oxalate. On the other hand, when pH is approximately 6, oxalate ion is the majority [51]. At the cytoplasmic pH of 7, oxalic acid also suffers deprotonation and exists as oxalate ion [2].

Chemically, oxalic acid is characterized as a dicarboxylic organic acid with low molecular weight, high acidity (pK_a1_ = 1.25, pK_a2_ = 4.27), and chelating and reducing abilities. Therefore, in plants, it plays a relevant role in many biological processes such as calcium homeostasis; pH regulation; plant growth, development, and protection; photosynthesis; and detoxification of heavy metals [2,3]. However, when in excess in plants because of a metabolic disorder, this will promote impairment of its functions and, thus, reduction of crop quality [2]. Many factors can influence oxalate accumulation in plants, such as growth, ripeness, variety, season, time of harvest, and cultivation conditions (e.g., use of nitrate fertilizer or soil conditions) [36,39,47,51].

The biosynthesis of oxalate in plants can result from different mechanisms, with glyoxylate, ascorbic acid, and oxaloacetate being the precursors of oxalate, an end product of their metabolisms. Therefore, there are three most-studied pathways: the glycolic acid/glyoxalic acid pathway, the ascorbic acid pathway, and the oxaloacetic acid pathway [2,51].

In addition to photosynthetic organisms, mammals can also produce oxalates in small amounts. In mammals, oxalate produced endogenously is a metabolite of ascorbate, hydroxyproline, glyoxylate, and glycine [3].

## 3. Analysis of Oxalates in Foods

According to the literature, various methods have been employed for the determination of oxalate in foods, including enzymatic assays, spectrofluorimetry, spectrophotometry, amperometry, electrochemistry, capillary electrophoresis, titration, GC, and HPLC [53]. All of these methods have advantages and disadvantages like their high sensitivity and specificity but also high costs and time-consuming and complex sample handling [54]. For example, samples require an additional step (esterification) for GC analysis [28,55]. Even though there are many others, this manuscript will only focus on HPLC and UV–Vis spectrophotometry, specifically on their extraction and analytical conditions. HPLC is the most recently referenced method used for the determination of oxalates because of its high sensitivity, accuracy, versatility, and reliability, despite requiring equipment that is expensive to purchase, repair, and maintain [29]. Conversely, despite its lower sensitivity, spectrophotometry is an inexpensive, simple, rapid, and accurate technique, using only a spectrophotometer as the main instrument [30,31].

### 3.1. Extraction Conditions

Accurate measurement of oxalate in foods is extremely dependent on its extraction, the first step in oxalate analysis. However, it seems to be difficult because of its extraction from plant tissue or its generation due to the oxidation of ascorbic acid during extraction [1].

Commonly, total oxalates (which include soluble and insoluble) are extracted with hydrochloric acid (HCl), whereas for soluble oxalates water is used [51]. However, a few papers describe different solutions, e.g., metaphosphoric acid [56], potassium phosphate buffer (pH 2.4) [57], or HCl with drops of octanol [58], for the extraction of total oxalates, and carbonate and sodium bicarbonate solution for the extraction of soluble oxalates [59,60]. Altunay et al. [30] also used HCl for the extraction of total oxalates and water for soluble oxalates; however, both extractions were conducted under ultrasonic power (300 W, 50 Hz).

The volume of the added extraction solution depends on the sample quantity, presenting a wide range of different values. Regarding other parameters, for HPLC analysis the hot extraction of total and soluble oxalates at 80 °C is more common, whereas for spectrophotometry 100 °C is more recurrent. For both methods, the most frequent time for extraction is 15 min. However, for HPLC, extraction times from 1–180 min (Table 1) are reported, whereas, for spectrophotometry, 15–1200 min is indicated (Table 2).

Additionally, some studies have shown the influence of modifying these extraction conditions. Hönow et al. [1] concluded that increasing extraction time from 30 to 180 min for the extraction of total oxalates resulted in oxalate generation and the increase was higher when treated at 100 °C (reflux) than at 80 °C (water bath). For soluble oxalates, results increased significantly after extraction at 80 °C compared to extraction at 21 °C and there were no significant differences between extractions of 15 or 30 min, proposing that soluble oxalate should be extracted with distilled water for 15 min at room temperature. In contrast, other authors suggested that room temperature might not be enough for the complete extraction of oxalates, leading to an underestimation [5]. This theory is also supported by Kusuma et al. [65] who considered that extraction temperatures above 65 °C are required for efficient extraction of total oxalates and pH should be at least 1. Therefore, the ideal temperature of oxalate extraction remains a controversial question because it can lead to oxalate generation due to in vitro conversion from precursors or failure to dissolve all pre-existing insoluble oxalates [1,5]. Concerning time, 15 min was considered to be the minimum for extraction and increasing it has not been associated with any advantage [65].

Frequently, further procedures after extraction mainly consist of filtration and centrifugation. However, some authors utilize more specific techniques. For example, prior to HPLC analysis, there have been reports of the use of solid-phase extraction (SPE) [35,55,69], purification with ion exchange [35], concentration [35,69], and, specifically for soluble oxalates, acidification with HCl to stabilize ascorbic acid which can be present and cause oxaloneogenesis at pH above 5, resulting in an overestimation [1,63,64,72]. For spectrophotometric methods, evaporation and subsequent redissolution for total oxalate analysis have been mentioned [58] and, once again, adjustment of pH, which is relevant for soluble oxalates [41,76,78].

Insoluble oxalates are also extracted during treatment with HCl, so their content is always calculated by the difference between total and soluble oxalates as Holloway et al. [83] suggested.

### 3.2. Methods’ Conditions

Despite having similar extraction conditions, chromatographic and spectrophotometric methods have different and specific features (Table 3 and Table 4).

#### 3.2.1. HPLC Conditions

Regarding HPLC conditions for the determination of oxalates (Table 3), ion exchange, ion exclusion, and reversed-phase columns are used. Considering ion exchange columns, one of the most referenced columns is IonPac AS4A [1,59,60,62,63,64,72]. Other authors have used different ones: propylamine anion exchange column (25 cm × 4.6 mm; 6 μm particle size), diethylaminoethyl (DEAE) anion exchange column (250 × 4.6 mm; 5 μm particle size), and Hamilton HA-X8.00 column with strong anion exchange resin in the sulfate form (255 × 5 mm; 7–10 μm particle size) [84]; Alltech All-Sep anion exchange column (100 × 4.6 mm; 7 µm particle size) [17] and Shodex IC SI-90 anion exchange column (250 × 4 mm; 9 μm particle size) filled with KanK-ASt (120 × 5 mm, 14 μm particle size) [71]. For ion exclusion, it seems that an ion exclusion column (300 × 7.8 mm) is frequently the selected one, either from Bio-Rad [32,34,40,61,70] or Rezex [65,66,67,68] brands. Regarding reversed-phase columns, the frequent use of 250 × 4.6 mm columns with different particle sizes has been reported [35,36,39,55,56,58,73,84], with 5 μm being the most common. Additionally, a radial compression column with C-18 or C-8 functionality (100 mm; 10 μm particle size) [84] has also been used. Some consumables listed in Table 3 may no longer be available in the market or have been replaced by columns with enhanced characteristics that contribute to the improvement of the analysis.

These columns are chosen considering different separation modes of HPLC. Ion chromatography is an effective method for the determination of oxalate ions because oxalic acid is a strong acid, giving away its protons and becoming negatively charged [71].

When using ion exclusion chromatography, the dissociated functional groups of the ion exchange resin present in the stationary phase have the same charge signal as the oxalate ion and, thus, it is repulsed and eluted [85]. In contrast, ion exchange chromatography is based on the exchange of oxalate ions with the counter-ions, which are anions of the ionic groups attached to the solid support being strongly retained [29]. Reversed-phase chromatography uses a non-polar stationary phase (e.g., C-18) with a polar mobile phase, being the most popular HPLC mode. The separation mechanism is based on polarity and hydrophobic/hydrophilic interactions between oxalates and these two phases. Furthermore, the use of a guard column, a smaller column applied before the analytical column to protect it from impurities present in samples and enhancing the lifetime of the main column, is commonly observed [29]. For the determination of oxalates, C-18 [55], amino [35], IonPac AG4A [59,60], and cation-H [32,34,40,65,66,67,68] guard columns are mentioned.

The main used type of elution for oxalate determination is undoubtedly isocratic. In a study using different elution programs for the separation of various organic acids, including oxalic acid, a gradient of (NH_4_)_2_SO_4_ and MgSO_4_ resulted in a rise in the baseline of the chromatograms [84]. Thus, using an isocratic elution promotes better results.

For the measurement of oxalates, there is a wide variety of mobile phases. For ion exchange columns, a carbonate and sodium bicarbonate solution for a conductivity detector [17,59,60,71] and an aqueous EDTA solution for amperometric detection are frequently utilized [1,63,64,72]. Some other mobile phases have been reported like 0.15 M NaH_2_PO_4_ (pH = 4.2); 0.30 M NH_4_H_2_PO_4_ adjusted to pH 6.5 with concentrated ammonium hydroxide; 0.5 M (NH_4_)_2_SO_4_ adjusted to pH 7.25 with ammonium hydroxide; 0.3 M (NH_4_)_2_SO_4_ containing 10% methanol and 0.1–1.5 M MgSO_4_ solutions for the gradient analysis [84]. When using ion exclusion columns, sulfuric acid is always the chosen mobile phase. However, Zulkhairi et al. [68] used a mixture of this acid with acetonitrile. For reversed-phase columns, dihydrogen phosphate (H_2_PO_4_^−^) is frequently used. This ion can be used alone [35,84] or combined in a mixture with tertiary butyl alcohol (TBA) [55] or tetrabutylammonium hydrogen sulfate [36,39] and it is frequently buffered with phosphoric acid (*circa* pH 2) [36,55,58,84]. Other authors used different solutions as mobile phase, for example, tetrabutylammonium chloride [73] and sulfuric acid [56]. In addition, buffer solutions have a big impact on the retention of analytes [29], so there is frequent use of these solutions to maintain a stable pH. For reversed-phase and ion exclusion columns, acidic buffers from approximately pH 2–3 are commonly used. In contrast, for ion exchange columns, higher pH values are allowed (*circa* pH 5).

The flow rate varies from 0.4–2 mL/min, with 1 mL/min or less being more common. When it is mentioned, column temperature ranges from room temperature to 80 °C. However, usually lower temperatures than 80 °C, such as 20 °C [58], 25 °C [65,66], 30 °C [73], 33 °C [71], or 50 °C, are used [58,68,70]. The most-reported injection volumes are 5 or 20 μL.

The most usual detectors are ultraviolet (UV) or ultraviolet–visible (UV–Vis), with 210 nm as the most common wavelength. In contrast, some authors report measurements at 206 [35,84], 215 [40,56], and 220 nm [39,55]. The choice of wavelength is usually made considering the maximum absorbance of analytes and, thus, maximum sensitivity. However, there are other parameters which are also taken into account for this choice, such as analysis time. Other used detectors are diode array detectors (DADs) or photodiode array (PDA) [68], which also detect absorption in the UV to Vis region but can scan the entire range [86]; refractive index (RI) detectors [84]; conductivity detectors [17,59,60,71]; and amperometric detectors [1,63,64,72]. These instruments are chosen depending on the type of HPLC analysis, considering its analytes. For example, authors who employed ion exchange chromatography for oxalate measurement have utilized conductivity detectors, whereas researchers who applied the HPLC–enzyme reactor method (HPLC-ER) used an amperometric detector. This last method is based on the chromatographic separation of oxalate combined with enzymatic conversion to hydrogen peroxide by oxalate oxidase and its amperometric detection [72,87].

#### 3.2.2. Spectrophotometric Conditions

Contrasting with HPLC, studies that describe spectrophotometric methods analyze much fewer samples in quantity and variety, whereas authors who applied HPLC present a large number of results of various foods. In the measurement of oxalates by spectrophotometry (Table 4), spinach and mushrooms are frequently studied. These observations might be due to HPLC’s ability for automatization and spectrophotometry’s laborious procedures when applied to a large number of samples.

Spectrophotometric conditions are quite peculiar. The studies analyzed for this matter include catalytic/kinetic methods. In other words, these procedures follow a spectrophotometric reaction which has some kind of oxalate intervention, either as a reagent [30,41,58,75,76,77], catalyst [31,37,38,74,78,79,80,81,82], or activator [54]. Some manuscripts mention other studies in which oxalate acts as an inhibitor [54,78]. These are indirect methods since they do not measure the absorbance of oxalate, but by measuring the absorbance of a substance within a system where oxalate has influence, it is possible to extrapolate oxalate content.

Most of these systems are redox reactions, using a wide range of different reagents (Table 4). For oxalate measurement, the most common type of redox reaction is oxidation in acidic media. However, there are a few reductions reported [58,75]. The use of potassium dichromate as an oxidant agent is frequently observed, but oxygen [77] and bromate [54] have also been reported. In contrast, oxalate has been used as a reducing agent [58,75].

In addition, other more complex procedures exist. For example, Matsubara et al. [77] studied a spectrophotometric method based on the oxidation of oxalate by oxygen in the presence of oxalate oxidase, forming hydrogen peroxide (H_2_O_2_) which forms a complex with TiO(tpypH_4_)^4+^ and TiO_2_(tpypH_4_)^4+^ that absorbs at 450 nm. Furthermore, Mo(VI) can form a stable complex with oxalate, [MoO_3_(Ox)]^2−^, which can have different forms in a solution in the pH range of 2–7, [Mo_2_O_5_(Ox)_2_]^2−^, or [Mo_2_O_5_(OH)(Ox)_2_]^3−^. Subsequently, this anionic complex associates with Toluidine blue (TBH^2+^) which has a maximum peak absorbance at 627 nm [30]. A new sensor material for solid-phase spectrophotometric determination of food oxalates was also developed. This method is based on the interaction of oxalates with a material of silica–titania xerogel with eriochrome cyanine R (Si-Ti/ECR) which causes sensor material discoloration, with absorbance being used as an analytical signal [41]. According to Tavallali et al. [76], the reaction of Reactive blue 4 (RB4)-Cu^2+^ with oxalate can be used for oxalate determination in food samples. The addition of oxalate to the RB4-Cu^2+^ complex increased the absorption band intensity at 607 nm and changed the color from sky blue to dark blue due to the regeneration of RB4 by the chelation of oxalate with Cu^2+^, since the binding constant of Cu^2+^ with oxalate is larger than that of Cu^2+^ with RB4.

Temperature, pH, and time of reactions are highly specific to each reaction, being parameters optimized before selecting the final procedure. The values of these parameters are chosen considering sensitivity and reproducibility [31,78]. Temperature, pH, and time of these reactions can range from approximately 20–90 °C, 3–7, and 8–60 min, respectively.

Reactions are monitored by measuring the absorbance of the reagents or products which are chromophore substances, such as crystal violet, Victoria blue, and brilliant cresyl blue, at maximum wavelength, *λ*_*max*_, the wavelength whose absorbance is maximum and producing maximum sensitivity. This measurement is always in the UV–*Vis* region (200–800 nm), mainly in the visible region (400–800 nm), since analyzed compounds are frequently colored and absorb this kind of light. For the construction of calibration graphs, oxalic acid as the standard solution was mainly used, but sodium oxalate [30,54,75,77,78,80] and potassium oxalate [58,76] were also reported.

By reading spectrophotometric methods for the determination of oxalate content, it can be concluded that validation is a frequent concern. Thus, linearity range, determination coefficient (r^2^), and limit of detection (LOD) are often presented parameters (Table 4).

**Figure 2:**
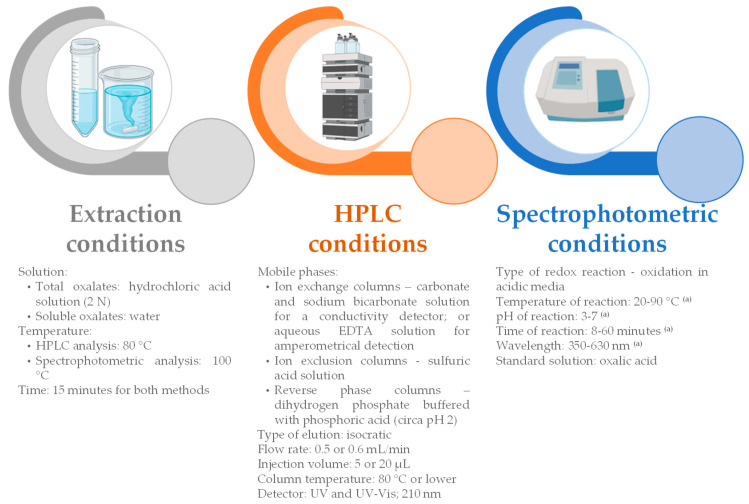
Summary of the most commonly used conditions for extraction for HPLC and spectrophotometry measurement of oxalate in foods. ^(a)^—These parameters are highly specific to each reaction (Table 4).

In Figure 2, a summary of the most commonly used conditions for extraction for HPLC and spectrophotometry analysis is provided. However, according to the literature, a wide variety of techniques and conditions are applied depending on the type of food, which makes it difficult to define a single method to measure oxalate in foods.

## 4. Oxalate Occurrence in Foods

Oxalate contents (mg/100 g FW) of various foods measured by chromatographic and spectrophotometric methods, such as in fruits, vegetables, mushrooms, legumes, pseudocereals, and aromatic plants, are collected in Table 5 in alphabetical order. Other studies were also taken into consideration; however, their oxalate contents were not included in this table because they were presented in dry weight and it was not possible to convert them into fresh weight owing to the lack of moisture or dry matter values [57,58,60,62,66,68].

In general, fruits are considered low-oxalate foods (<30 mg total oxalate/100 g FW), except for star fruit (160 [35] and 295.4 [1] mg total oxalate/100 g FW), elderberry (72.1 mg total oxalate/100 g FW), and dried fig (95.1 mg total oxalate/100 g FW) [1]. However, it is to be noted that for elderberry and dried fig, the majority of oxalate content is insoluble (72.1 and 89.6 mg/100 g FW, respectively) and, thus, less harmful [1].

Regarding vegetables, there is much more variety in the values of oxalate content, ranging from not detected to high amounts of oxalate. For example, raw New Zealand spinach and typical spinach have been reported as extremely high in oxalates with values of 1764.7 [32] and 329.6–2350 mg total oxalate/100 g FW [32,33,34,35,36,37,38,39], respectively. Soluble oxalate concentration in *Spinacia oleracea* has been studied during the cultivation season. Oxalate content was higher in winter (1092.9 mg soluble oxalate/100 g FW) and lowest in fall (614.9 mg soluble oxalate/100 g FW), indicating that higher oxalate content is related to a longer growing period since this compound is an end product of some metabolisms and it increases in the vacuole with plant aging [73]. Also, rhubarb contained high oxalate content (1235 mg total oxalate/100 g FW) [1] as well as Swiss chard with 874 [1] and 1458.1 [40] mg total oxalate/100 g FW, having more in leaves than in stems [32], and sorrel with 1079 mg soluble oxalate/100 g FW [41]. Taro leaves yielded 300.2–721.9 mg total oxalate/100 g FW, depending on the type of cultivar and processing techniques: baking increased oxalate content compared to raw samples, due to concentrating effects, whereas baking with milk (a source of calcium) decreased oxalate content, especially soluble [34]. This decrease happens because oxalate ions can bind with calcium, precipitating and reducing soluble oxalate [45]. Conversely, there were some vegetables with small amounts of oxalate or it was even undetected, e.g., cabbage, broccoli, cauliflower, cucumber, kale, and pumpkin (Table 5).

Regarding legumes, soybean is considered high in oxalates, ranging between 124 and 497 mg total oxalate/100 g FW, depending on the kind of sample analyzed [36,61,63]. For different types of beans, oxalate values ranged between 13.9 and 547.9 mg total oxalate/100 g FW [1,36,61,63], whereas chickpeas [63] and lentils [1,63] yielded low oxalate content (<24 mg total oxalate/100 g FW) and oxalate in cowpea was not detected [61].

In the pseudocereals category, amaranth is considered a high-oxalate food (1510.8 mg total oxalate/100 g FW) [40] as well as green amaranth (*circa* 1940 mg total oxalate/100 g FW) and purple amaranth (*circa* 1354 mg total oxalate/100 g FW) [33,34].

For aromatic plants, two different species of parsley, water dropwort (also known as water celery), and coriander yielded 136 [1] and 270.7 [40], 93 [40], and *circa* 41 [33,34] mg total oxalate/100 g FW, respectively. Licorice was the highest-oxalate food reported in this review with 3569.3 mg total oxalate/100 g FW [63]. In contrast, arugula, cress, garlic, and green onion were some examples of aromatic plants which did not contain detectable oxalate (Table 5).

Differences between oxalate values for the same food, as observed for beans, lettuce, parsley, mushrooms, or spinach, can vary according to growth, ripeness, climate, region, soil conditions, and time of harvest. In addition to these conditions, which are harder to control, are sample preparation, which can lead to oxalate generation or its incomplete extraction, and analytical methods with different features that can have an impact on oxalate results [1,6,36,65].

It is important to consider the usual amount of consumption of these foods. For example, some aromatic plants, like parsley, contained high oxalate values but their daily intake is naturally much less than 100 g. Also, the type of consumption has to be taken into account because some foods are not generally eaten in the form in which they were analyzed (e.g., raw beans or mushrooms). It is well known that the same food prepared differently (raw, boiled, baked, fried, etc.) can lead to different oxalate results. The reported values for potato (*Solanum tuberosum*) are a great example to evidence the influence of cooking techniques on oxalate content [1]. In general, boiling has been associated with decreased oxalate content, especially soluble oxalates, due to its leaching and thermal degradation [45,47], as observed in spinach, New Zealand spinach, red and white beans, soybean, and rhubarb (Table 5).

## 5. Health Implications of Oxalates

Oxalate has been a concern for human health due to its antinutritive effects and potential nephrotoxicity for a long time [42,43]. In 1989, a fatality from oxalic acid poisoning was reported. A 53-year-old man, with other conditions, had eaten a sorrel soup with 6–8 g of oxalic acid [88]. A lethal dose of oxalic acid for adults was estimated as 10–15 g, although the ingestion of 4–5 g of oxalate was considered the minimum dose able to cause death [51,88]. As antinutrients, oxalates restrict the bioavailability of some nutrients since they can bind to minerals, like calcium, magnesium, or iron, reducing their absorption and use [3,44].

**Figure 3:**
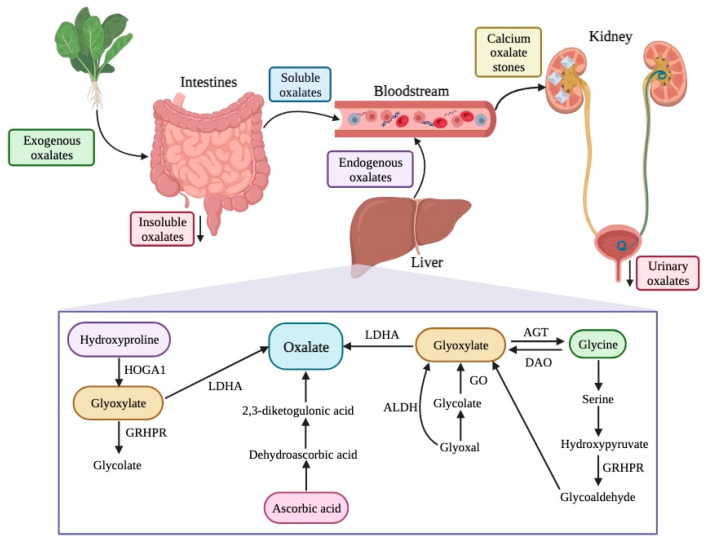
Sources, endogenous pathways, and excretion of oxalates in the human body. HOGA1—4-hydroxy-2-oxoglutarate aldolase type 1; GRHPR—glyoxylate reductase/hydroxypyruvate reductase; LDHA—lactate dehydrogenase A; ALDH—aldehyde dehydrogenase; GO—glycolate oxidase; AGT—alanine/glyoxylate aminotransferase; DAO—D-amino acid oxidase.

The sources of oxalates in our body can be exogenous or endogenous (Figure 3). Exogenous oxalate sources are mainly plant foods, like vegetables, grains, legumes, and fruits, among others. When these types of foods are ingested, oxalate is absorbed in multiple parts of our gastrointestinal tract, namely the stomach, small intestine, and large intestine. However, the absorption depends on its availability, among other individual features. Insoluble oxalates are excreted in feces since they are less bioavailable and, therefore, pose a lower health risk. In contrast, soluble oxalates are absorbed through the intestines and colon (5–10% of ingested oxalate, under normal conditions), going into the bloodstream.

Since absorption of oxalates is related to the amount of soluble oxalates, which are more bioavailable, a simultaneous consumption of oxalate with calcium or magnesium can reduce its bioavailability and absorption due to the formation and fecal excretion of insoluble salts, lowering the health risk [1,45,47,52,89]. It has been reported that men with less than 755 mg/day of calcium intake had a higher risk of kidney stone formation, whereas men with a median calcium intake or above had a lower risk [3]. Therefore, dietary calcium intake has been inversely associated with kidney stone formation [3,52,90,91].

Also, it has been observed that intestinal absorption of oxalates in individuals with a history of stone formation was expressly higher than in healthy individuals (9.2% and 6.7%, respectively) [1]. Gastrointestinal health influences oxalate absorption as well, with soluble oxalate being excessively absorbed due to intestinal malfunction.

Despite these facts, oxalate is not typically consumed daily in high concentrations and there are other constituents in foods which have a protective role against kidney stone formation, such as phytate, potassium, calcium, and antioxidant phytochemicals like polyphenols [3]. Also, boiling, steaming, soaking, and processing with calcium sources are some procedures to reduce the content of soluble oxalates, the most harmful oxalates [45,52].

Concerning the endogenous production of oxalates, the liver is the primary source. There are different pathways for oxalate production, including the metabolism of protein (through amino acids, like tyrosine, tryptophan, phenylalanine, and hydroxyproline), ascorbic acid, and precursors of oxalate (such as L-glycerate glycollate and glyoxylate) [92,93]. Glyoxylate is an important intermediary product in several reactions and, for its metabolization into oxalate, enzymes like glycolate oxidase and lactate dehydrogenase are needed.

Free oxalates are delivered to the kidney and can be excreted, increasing urinary oxalates, or can chelate with calcium ions there, resulting in calcium oxalate crystals, which can cause serious health issues like kidney stones, also known as nephrolithiasis or urolithiasis (Figure 3) [3,45,46,47,48,91].

This crystallization in the kidney infiltrates vessel walls and can lead to renal tubular obstruction, vascular necrosis, and hemorrhage, which can cause anuria, uremia, electrolyte disturbances, or even rupture and kidney failure [48,51]. Calcium oxalate and its relationship with kidney stone formation have been amply studied, with calcium oxalate being one of the most common types of human kidney stone reported, followed by calcium phosphate [46,51,90,94,95].

Hyperoxaluria is a metabolic disease that leads to excessive urinary oxalate excretion (>40–45 mg/day) [89,96], being an indicator of possible kidney stone formation [91]. The most reliable way to assess daily oxalate intake is through 24 h urine collection; however, there are also food frequency questionnaires whose credibility is debated [89,91].

Hyperoxaluria can be divided into primary hyperoxaluria (PH1) and secondary hyperoxaluria (PH2). PH1 is a group of rare autosomal recessive diseases that negatively affect key enzymes of oxalate metabolism, leading to an overproduction of oxalates in the liver [50]. When renal function declines and excess oxalate exists in the bloodstream, a phenomenon known as systemic oxalosis occurs and calcium oxalate crystals deposit in various organs, tissues, and bones [50,96,97]. Severe damage in the eyes, joints [98], myocardium, skin [99], oral tissues [96], and bone marrow [49] is reported. Oxalate can also be associated with acute kidney injury, a tubular obstruction due to calcium oxalate crystal deposition, and with chronic kidney disease progression, but further studies are necessary [89].

Patients with hyperoxaluria, especially PH1, from a clinical point of view, frequently present severe bone pain, pathological fractures, and bone deformations. This is frequently associated with the fact that calcium oxalate crystals may deposit within bones, tendons, cartilage, and synovium, causing oxalate arthritis. Then, the calcium oxalate crystals may enter into the synovial fluid, where an inflammatory response will arise, leading to joint effusions and arthralgias [100,101].

PH2 results from increased intestinal absorption of dietary oxalates and can also lead to excessive urinary oxalate [48,51,102]. A high intake of foods rich in oxalate, enteric hyperoxaluria, oxalate-degrading mechanisms, and SLC4 and SLC26 ionic exchangers are linked with PH2. Dietary oxalate plays an important role in PH2, contributing up to 72% of the urinary oxalate excreted [17]. Enteric hyperoxaluria is a form of PH2 that is linked with malabsorption syndromes due to disease or resection of the gastrointestinal tract. In foods, oxalate is usually complexed with calcium, resulting in insoluble oxalate, which is difficult to absorb. Nevertheless, in fat malabsorption conditions, the amount of free oxalates can increase, due to the capacity of free fats to bind calcium. Therefore, PH2 is linked with several conditions that cause fat malabsorption, such as inflammatory bowel disease, celiac disease, short bowel syndrome, and bariatric surgery, among others [100].

The gut microbiome plays an important role since some bacterial species can degrade oxalate to obtain carbon and energy and therefore reduce the concentration of oxalates in blood and urine, minimizing the formation of kidney stones [91,92,93,103,104].

The gut microbiota is usually similar between individuals; however, it can be affected by the age of individuals, by the diet, and by the use of antibiotics, among other factors. Probiotics are defined as “live microorganisms which, when administered in adequate amounts, confer a health benefit on the host” and are being abundantly used as preventive therapeutic agents for several diseases, since they have been implicated in the stabilization of gut microbiota and enhancement of immune responses [105].

The best-known oxalate-degrading microorganism is *Oxalobacter formigenes*, but others are also able to degrade oxalate into carbon dioxide and formate, namely *Escherichia coli*, *Bifidobacterium* spp., and *Lactobacillus* spp. [93,104]. *O. formigenes* is a Gram-negative anaerobic bacterium isolated from human feces and other animals that utilizes intestinal oxalate as a carbon source, through formyl-CoA transferase and oxalyl-CoA decarboxylase enzymes, and metabolizes the oxalate into carbon dioxide and formate, contributing to regulating oxalate homeostasis [103]. However, its application as a probiotic is limited due to its nutritious requirements, but also because it has less colonization ability and is sensitive to the use of certain antibiotics and drugs [106]. Moreover, the therapeutic use of *O. formigenes* can be compromised, for example, in patients with PH1 and patients with cystic fibrosis. To date, the best conditions (pH, sugar concentration), as well as the adequate amount of these supplements, are not clear and more research is still needed [93].

## 6. Conclusions

Various methods have been employed for the determination of oxalate in foods. Particularly, extraction and analytical conditions of HPLC and UV–*Vis* spectrophotometry were reviewed. Despite having different features, both methods have similar extraction procedures. Among other extraction parameters, temperature remains a controversial question because it can lead to oxalate formation from precursors or failure to dissolve all pre-existing insoluble oxalates. Furthermore, a considerable quantity of different HPLC and spectrophotometry methods were gathered and analyzed, concluding that there is a huge variety of procedures.

This review also compared the oxalate content (mg/100 g FW) of a wide range of foods, measured by HPLC and spectrophotometry. The results showed that spinach, New Zealand spinach, rhubarb, Swiss chard, taro leaves, sorrel, soybean, amaranth, parsley, and licorice contained high oxalate levels and can be considered high-oxalate foods, especially some green leafy vegetables. In contrast, others can be referred to as low-oxalate foods: fruits (except for star fruit, elderberry, and dried fig), cabbage, broccoli, cauliflower, cucumber, kale, pumpkin, chickpeas, lentils, cowpea, arugula, cress, garlic, and green onion. Nevertheless, there are some procedures to reduce oxalate, in particular soluble oxalate, such as boiling, steaming, soaking, and processing with calcium sources.

Despite a clear relationship between dietary oxalate, calcium oxalate, and kidney stone risk, the connection might be more complex than previously thought due to the impact of cooking techniques, calcium intake, endogenously produced oxalate, and gastrointestinal health. Foods which contain oxalates, such as fruits and vegetables, have a wide range of beneficial compounds that might outweigh possible negative implications on human health. Additionally, systemic oxalosis does not seem to be related to dietary oxalate, but to previous pathologic conditions of individuals such as primary hyperoxaluria. Hence, regular consumption of high-oxalate foods by healthy individuals as a part of a balanced and diversified diet does not appear to cause health issues if daily consumption is from 50–200 mg/day, whereas for individuals susceptible to kidney stone formation dietary modification is crucial for its prevention. For these individuals, it is recommended to limit the consumption of high-oxalate foods to less than 40–50 mg oxalate/day since they can present a health threat in these cases.

## Figures and Tables

**Table 1:** Extraction conditions for quantification of oxalates in foods by high-performance liquid chromatography.

Oxalates	Matrix	Sample Amount (g)	Extraction Conditions					Further Procedures before Injection	References
			Solution	Volume (mL)	pH	Temp. (°C)	Time (min)		
Total	Rhubarb petioles	25	HCl (1 N)	200	-	100	15	Filtration and SPE (Sep-Pak cartridges) regenerated with 4 mL of methanol and 2 mL of water. The first 2 mL was discarded and the rest used for HPLC analysis.	[55]
	Carambola and spinach	100	HCl (3 N)	100	-	-	1	Filtration with Buchner funnel and two extractions by stirring each time with 50 mL of water or 3 N HCl. Concentration to 100 mL at 30 °C under reduced pressure (20 mmHg). SPE (C-18 Sep-Pak cartridge) pretreated with 2 mL of acetonitrile and 5 mL of water. The first 2 mL of eluate was discarded and the rest was filtered through a 0.45 μm Millipore filter. Purification of some samples with ion exchange prior to HPLC analysis.	[35]
	Vegetables and spices	1	HCl (2 N)	50	0.09	80	15	Centrifugation at 3000 rpm and filtration of 10 mL of the supernatant through a 0.45 mm cellulose acetate membrane.	[32,33]
	Vegetables, cereal grains, and legume seeds				1				[61]
	Spinach	1	HCl (0.05 N)	-	-	-	-	-	[62]
			HCl (0.5 N)	5 (+5 deionized water)	-	100	20	Centrifugation at 12,000× *g* for 10 min. Supernatant removed and the volume increased to 20 mL with deionized water. Filtration with a filter with pore size 0.22 μm.	[39]
	Fruits, vegetables, beverages, spices, herbs, nuts, cereals, algae, and mushrooms	2	HCl (25 %)	4	-	80	30 and 180	Filtration of 1 mL of the solutions.	[1]
						100			
			HCl (2 N)			100			
						21	15		
							30		
						80	15		
							30		
	Cereal, cereal products, and plants of the Fabaceae, Convolvulaceae, and Malvaceae families	2	HCl (2 N)	4	-	21	15	Filtration.	[63,64]
	Spinach		HCl	40	0.93–5.81	25–95	15–120	Cooled by standing in running cold water (11.5 °C) for 5 min. Volume made up to 100 mL with the extraction solution. Filtration of the sample solutions using a 0.45 μm syringe filter into 1 mL glass vial.	[65]
	Taro leaves and corms and Indian vegetables	3 or 0.5	HCl (2 N)	50	0.7	80	15	Centrifugation at 3000 rpm and filtration of 10 mL of the supernatant through a 0.45 mm cellulose acetate membrane.	[34]
	Spinach	0.3	HCl (0.2 N)	40	-	80	15	Centrifugation at 2889× *g* for 15 min. Filtration of the supernatant through a 0.45 mm cellulose nitrate filter.	[66]
	Green juices with spinach and other vegetables and fruits	5					20	The extracts were allowed to cool and then made up to 100 mL, in a volumetric flask, with HCl (0.2 N).	[67]
	Korean vegetables		HCl (2 N)	40 (+20 after homogenization)	-	80	15	Filtration with filter paper. Dilution with deionized water (90 mL). Filtration with 0.45 μm regenerated cellulose microfilters.	[40]
	Pakistani vegetables and beans	5 (mixed with 5 mL of distilled water)	HCl (2 N)	50	-	-	-	Centrifugation at 5000 rpm for 20 min and the supernatants were transferred to 100 mL volumetric flasks and made up to final volume with distilled water.	[36]
	Taro corms	0.5	HCl (2 N)	20	-	80	15	The extracts were cooled at room temperature and made up to 50 mL with HCl (2 N). Centrifugation at 2889 rpm for 15 min. Separation and filtration of the supernatant through a 0.45 mm cellulose nitrate membrane.	[68]
	Mexican vegetables		HPO_3_ (45 g/L)	25	-	-	15	Centrifugation (30 min, 8960× *g*) and the supernatant made up to 25 mL with metaphosphoric acid.	[56]
	Ethiopian collard greens and mango	4	HCl (2 N)	50	-	-	50 (at 250 rpm)	The solution was removed from the shaker and 50 mL HPLC grade H_2_O was added. Filtration through 0.45 μm syringe filter. The filtrate was transferred into a 2 mL vial.	[58]
	Various foods	2 to 3	HCl (0.2 N)	-	-	60	60	Centrifugation (15,000× *g*; 5 min) and filtration through 25 mm diameter 0.2 µm PTFE filter.	[17]
	Spinach and kale	-	Potassium phosphate buffer (0.2 M)	-	2.4	-	-	-	[57]
Soluble	Carambola, spinach, spinach products, and New Zealand spinach	100	Water	100	-	-	1	Filtration with Buchner funnel and two extractions by stirring each time with 50 mL of water or 3 N HCl. Concentration to 100 mL at 30 °C under reduced pressure (20 mmHg). SPE (C-18 Sep-Pak cartridge) pretreated with 2 mL of acetonitrile and 5 mL of water. The first 2 mL of eluate was discarded and the rest was filtered through a 0.45 μm Millipore filter. Purification of some samples with ion exchange prior to HPLC analysis.	[35,69]
	Spinach	0.5	Carbonate (1.8 mM) and sodium bicarbonate (1.7 mM) solution	50	-	-	-	-	[60]
	Fresh vegetables					25	30		[59]
	Taro corms		Deionized water	20	-	80	15	The extracts were cooled at room temperature and made up to 50 mL with deionized water. Centrifugation at 2889 rpm for 15 min. Separation and filtration of the supernatant through a 0.45 mm cellulose nitrate membrane.	[68]
	Mexican vegetables		Distilled water	25	-	-	15	Centrifugation (30 min, 8960× *g*) and the supernatant was made up to 25 mL with metaphosphoric acid.	[56]
	Vegetables and spices	1	Distilled water	50	6.90	80	15	Centrifugation (3000 rpm) and filtration of 10 mL of the supernatant through a 0.45 mm cellulose acetate membrane.	[32,33]
	Vegetables, cereal grains, and legume seeds				5.50				[61]
	Samples of herbal, green, oolong, and black teas	-	Distilled water	50	6.90	80	15	Each sample of tea was filtered through a 0.45 mm cellulose acetate membrane syringe filter.	[70]
	Taro leaves and corms and Indian vegetables	3 or 0.5	Distilled water	50	6.50	80	15	Centrifugation at 3000 rpm and filtration of 10 mL of the supernatant through a 0.45 mm cellulose acetate membrane.	[34]
	Fruits, vegetables, beverages, spices, herbs, nuts, cereals, algae, and mushrooms	2	Distilled water	4	-	21	15	Filtration and acidification by adding HCl (50 μL/mL, 2 N) to stabilize ascorbic acid.	[1]
							30		
						80	15		
							30		
						21	15		
	Green and black tea	2	Water	-	-	100	6	Filtration of the obtained extract through a disposable 0.45 μm filter. Dilution 10 times.	[71]
	Cereal, cereal products, and plants of the Fabaceae, Convolvulaceae, and Malvaceae families		Distilled water	4	-	21	15	Filtration and acidification by adding HCl (50 μL/mL, 2 N) to stabilize ascorbic acid.	[63,64]
	Korean vegetables	5	Water	40 (+20 after homogenization)	-	80	15	Filtration with filter paper. Dilution with deionized water (90 mL). Filtration with 0.45 μm regenerated cellulose microfilters.	[40]
	Green juices with spinach and other vegetables and fruits		Nanopure II water	40	-	80	20	The extracts were allowed to cool and then made up to 100 mL, in a volumetric flask, with Nanopure II water.	[67]
	Pakistani vegetables and beans	5 (mixed with 5 mL of distilled water)	Distilled water	50	-	-	-	Centrifugation at 5000 rpm for 20 min and the supernatants were transferred to 100 mL volumetric flasks and made up to final volume with distilled water.	[36]
	Spinach	0.3	Nanopure water	40	-	80	15	The extracts were allowed to cool and then transferred into 100 mL volumetric flasks and made up to final volume. Centrifugation at 2889× *g* for 15 min. The supernatant was filtered through a 0.45 mm cellulose nitrate filter.	[66]
	Alcoholic and non-alcoholic beverages	1.75 to 7	Water	200 or 150	-	70	5	Filtration of 4 mL of samples and acidification with 50 μL HCl (2 N).	[72]
	Spinach	-	Deionized water	10 times the volume of fresh weight	-	RT	5	Filtration through filtrate paper (ADVANTEC no. 3; Advantec, Tokyo) on ice. Further filtration using the spin column Ultra-free-MC 0.45 μm PTFE membrane (Millipore, Billerica, Mass.) and centrifugation at 5000× *g* for 60 min at 4 °C. Storage of samples at –20 °C. Frozen filtrates were thawed on ice, diluted to 10× with deionized water, and transferred to a disposable vial (S/T micro vial; Tomsic, Tokyo).	[73]
	Spinach	1	Deionized water	5 (+5 after cooling)	-	100	20	Centrifugation at 12,000× *g* for 10 min. The supernatant was removed and the volume made up to 20 mL with deionized water. Filtration with a filter with pore size 0.22 μm.	[39]

RT—room temperature; Temp.—temperature; “-“—information not available.

**Table 2:** Extraction conditions for quantification of oxalates in foods by spectrophotometry.

Oxalates	Matrix	Sample Amount (g)	Extraction Conditions				Further Procedures before Analysis	References
			Solution	Volume (mL)	Temp. (°C)	Time (min)		
Total	Cress, dill, parsley, cauliflower, broccoli, celery, black cabbage, red radish, lettuce, and leek	3	HCl (0.2 N)	30	60	15	The mixture of the sample with HCl was degassed and digested under ultrasonic power (300 W, 50 Hz). It was allowed to cool and then filtered by using a membrane filter of 0.45 μm into a 100 mL volumetric flask. The final volume was diluted to 100 mL with ultrapure water before analysis.	[30]
	Ethiopian collard greens, cabbage, lettuce, beetroot, pineapple, and mango	1	(a) Water, (b) HCl (6 N), (c) octanol	(a) 150, (b) 27.5, (c) 2 drops	100	25	The mixture was cooled, transferred to a 250 mL volumetric flask, and the volume completed. Filtration through Whatman 541 filter paper. Evaporation of 10 mL of this filtrate at 40–45 °C in a vacuum oven and redissolution in 10 mL of 0.01 M H_2_SO_4_.	[58]
	Spinach	0.50	HCl (2 N)	20	-	-	Centrifugation at 2500 rpm for 6 min and the supernatant was filtered through Whatman No. 1 paper. The residue retained by the filter was treated twice with 10 mL of HCl (2 N) and the combined filtrates were diluted to 250 mL with water.	[37]
		5	HCl (2 N)	20	-	-		[38]
	Spinach and mushroom	5	HCl (2 N)	20	-	-	Centrifugation of the suspension at 2500 rpm for 6 min. Filtration of the supernatant through filter paper (Whatman No. 1). The residue retained by the filter was treated twice with 10 mL of HCl (2 N) and then filtered. The combined filtrates were mixed and diluted to 100 mL with water.	[74]
Soluble	Spinach and mushroom	2.5 or 25	Water	-	100	20	The suspension, previously cooled, was centrifuged and then filtered through filter paper (Whatman No. 1). Dilution of the filtrate to 1000 cm^3^ and 2.0 cm^3^ of the sample solution was used in the proposed method.	[54]
	Cress, dill, parsley, cauliflower, broccoli, celery, black cabbage, red radish, lettuce, and leek	3	Ultrapure water	30	60	15	The mixture of the sample with water was degassed and digested under ultrasonic power (300 W, 50 Hz). It was allowed to cool and then filtered by using a membrane filter of 0.45 μm into a 100 mL volumetric flask. The final volume was diluted to 100 mL with ultrapure water before analysis.	[30]
	Sorrel, spinach, parsley, ginger, and black pepper	10	Distilled water	100	100	15	Filtration after cooling. The solution pH was adjusted to 3.0 and the final solution was diluted to 100 mL. Dilution five times.	[41]
	Tap water	50	-	-	100	20	-	[75]
	Spinach and mushroom	3 or 15	Water	Necessary to dilute to 100 mL in a calibrated flask	100	45	After cooling, the filtration of the suspension was carried out through Whatman No. 1 filter paper. Dilution to 250 mL. Adjustment of the pH to about 10 by dropwise addition of NaOH (0.1 N). The solution was centrifuged (MS-3400 centrifuge; Cole-Parmer, St. Neots, UK) at 1492× *g* for 5 min. After the neutralization of the solution with HCl (0.1 N), it was diluted in a 100 mL volumetric flask.	[76]
	Vegetables, beverages, and fruits	20	Distilled water	60 (+HCl to adjust to pH 2–3)	50	15	After cooling to room temperature and adjusting to pH 3.0 with potassium hydroxide, the mixtures were transferred to 100 mL volumetric flasks and they were made up with distilled water. Filtration through membrane filters (0.45 μm).	[77]
	Spinach and mushrooms	3 or 15	Water	-	100	45	The suspension was filtered twice through filter paper (Whatman No. 1) and the filtrate was diluted to 250 mL. Adjustment of the pH to about 10 by dropwise addition of 0.10 N sodium hydroxide solution. Centrifugation at 2000 rpm for 5 min. The resulting solution was decanted, neutralized with HCl (0.10 N), and diluted in a 100 mL volumetric flask. Then, 1.0 mL of the solution obtained was used for the proposed method.	[78]
	Spinach, mushroom, and kidney bean	5 to 10	Water	250	-	10	Centrifugation at 2500 rpm for 5 min. Filtration of the supernatant dryly through Whatman No. 1 paper.	[79]
	Spinach	5	Water	-	100	30	Filtration through a filter paper, after being cooled. The filtrate was diluted to 250 mL and 5 mL of this solution was used to determine oxalate.	[80]
	Spinach	5	Water	Necessary to dilute to 50 mL in a volumetric flask	-	1200	Centrifugation of the suspension at 2500 rpm for 10 min. Filtration of the supernatant through Whatman No. 1 paper.	[81]
	Spinach	5	Water	Necessary to dilute to 50 mL in a volumetric flask	-	-	Centrifugation of the suspension at 2500 rpm for 5 min. Filtration of the supernatant through Whatman No. 1 paper.	[31]
	Beetroot, spinach, and mushroom	50	Water	-	100	50	The mixture was cooled and filtered through a membrane filter.	[82]

“-“—information not available.

**Table 3:** High-performance liquid chromatography conditions for the analysis of oxalates in foods.

Matrix	Analytes	Chromatographic Conditions	Validation Data	References
Rhubarb petioles	Total oxalates	Column: LiChrosorb RP-8 (250 × 4.6 mm; 10 μm particle size) Guard column: LC C-18 guard column Detector (λ, nm): UV (220) Mobile phase: 0.5% KH_2_PO_4_ and 0.005 M TBA buffered at pH 2.00 with orthophosphoric acid Type of elution: Isocratic Injection volume (µL): 5 Flow rate (mL/min): 2 Column temp: (°C): - Run time (min): 7	Linearity range: 0–0.40 mg/mL Determination coefficient (r^2^): - LOD: < 0.001 mg/mL LOQ: -	[55]
Carambola and spinach	Total oxalates	Column: Zorbax amine (250 × 4.6 mm; 7 μm particle size) Guard column: Amino guard column (10 μm particle size) Detector (λ, nm): UV (206) Mobile phase: Buffer solution of aqueous NaH_2_PO_4_ (0.15 M) at pH 2.4 Type of elution: Isocratic Injection volume (µL): 20 Flow rate (mL/min): 1.1 Column temp: (°C): - Run time (min): -	Linearity range: - Determination coefficient (r^2^): - LOD: - LOQ: -	[35]
-	Organic acids (e.g., oxalic acid)	Columns: (1) Radial compression column with C-18 or C-8 functionality (100 mm; 10 μm particle size); (2) C-8 analytical columns of two manufacturers (250 × 4.6 mm, 6 and 10 μm particle size); (3) propylamine anion exchange column (25 cm × 4.6 mm; 6 μm particle size) (4) diethylaminoethyl (DEAE) anion exchange column (250 × 4.6 mm; 5 μm particle size); and (5) Hamilton HA-X8.00 column with strong anion exchange resin in the sulfate form (255 × 5 mm; 7–10-μm particle size) Guard column: - Detector (λ, nm): RI and UV (254 and 206) Mobile phase: (1–2) 2% NH_4_H_2_PO_4_ adjusted to pH 2.4 with phosphoric acid; (3) 0.15 M NaH_2_PO_4_ (pH = 4.2); (4) 0.30 M NH_4_H_2_PO_4_ adjusted to pH 6.5 with concentrated ammonium hydroxide; (5) 0.5 M (NH_4_)_2_SO_4_ adjusted to pH 7.25 with ammonium hydroxide; 0.3 M (NH_4_)_2_SO_4_ containing 10% methanol and 0.1–1.5 M MgSO_4_ solutions (for the gradient analysis) Type of elution: (1–5) Isocratic and (5) linear gradient Injection volume (µL): 20 Flow rate (mL/min): 1; 1.5 and 2 Column temp: (°C): (columns 1 to 4) RT and (column 5) 80 Run time (min): -	Linearity range: - Determination coefficient (r^2^): - LOD: - LOQ: -	[84]
Spinach	Soluble oxalates	Column: IonPac AS4A (250 × 4 mm; 15 µm particle size) Guard column: IonPac AG4A Detector (λ, nm): Conductivity Mobile phase: Na_2_CO_3_ (1.8 mM) and NaHCO_3_ (1.7 mM) solution Type of elution: Isocratic Injection volume (µL): - Flow rate (mL/min): - Column temp: (°C): - Run time (min): -	Linearity range: - Determination coefficient (r^2^): - LOD: - LOQ: -	[60]
Vegetables	Soluble oxalates	Column: IonPac AS4A (250 × 4 mm; 15 µm particle size) Guard column: IonPac AG4A Detector (λ, nm): Conductivity Mobile phase: Na_2_CO_3_ (1.8 mM) and NaHCO_3_ (1.7 mM) solution Type of elution: Isocratic Injection volume (µL): - Flow rate (mL/min): 2 Column temp: (°C): - Run time (min): -	Linearity range: - Determination coefficient (r^2^): - LOD: - LOQ: -	[59]
Spinach	Total oxalates	Column: Dionex IonPac AS4A-SC (250 × 4 mm; 13 µm particle size) Guard column: - Detector (λ, nm): - Mobile phase: - Type of elution: - Injection volume (µL): - Flow rate (mL/min): - Column temp: (°C): - Run time (min): -	Linearity range: - Determination coefficient (r^2^): - LOD: - LOQ: -	[62]
Spinach, swiss chard, broccoli, carrot, parsnip, rhubarb stalks, and beetroot	Total and soluble oxalates	Column: Bio-Rad Aminex ion exclusion HPX-87H (300 × 7.8 mm; 9 µm particle size) Guard column: Aminex Cation-H guard column Detector (λ, nm): UV–Vis (210) Mobile phase: H_2_SO_4_ (0.0125 M) filtered through a 0.45 µm membrane and degassed using vacuum Type of elution: Isocratic Injection volume (µL): 5 Flow rate (mL/min): 0.5 (0.1 prior to use and in between sample sets) Column temp: (°C): RT Run time (min): -	Linearity range: 0.01–0.2 mg/mL Determination coefficient (r^2^): 0.999 (total) and 0.986 (soluble) LOD: - LOQ: -	[32]
Various foods	Soluble oxalates	Column: Alltech All-Sep anion exchange column (100 × 4.6 mm; 7 µm particle size) Guard column: - Detector (λ, nm): Conductivity Mobile phase: Na_2_CO_3_ (0.9 mM) and NaHCO_3_ (0.85 mM) Type of elution: Isocratic Injection volume (µL): - Flow rate (mL/min): 1.2 Column temp: (°C): - Run time (min): -	Linearity range: 0.5 × 10^−9^–1 × 10^−8^ mg/mL Determination coefficient (r^2^): 0.990 LOD: - LOQ: 0.2 mg/100 g	[17]
Samples of herbal, green, oolong, and black teas	Soluble oxalates	Column: Bio-Rad Aminex ion exclusion HPX-87H (300 × 7.8 mm; 9 µm particle size) Guard column: - Detector (λ, nm): UV (210) Mobile phase: H_2_SO_4_ (0.0125 M) Type of elution: Isocratic Injection volume (µL): 5 Flow rate (mL/min): 0.5 Column temp: (°C): 50 Run time (min): -	Linearity range: - Determination coefficient (r^2^): - LOD: - LOQ: -	[70]
Fruits, vegetables, beverages, spices, herbs, nuts, cereals, algae, and mushrooms	Total and soluble oxalates	Column: Dionex IonPac AS4A anion exchange column (250 × 4 mm; 15 µm particle size) Guard column: - Detector (λ, nm): Amperometric Mobile phase: 2.0 g EDTA/L distilled water adjusted to pH 5.0 by adding 15 mL of 0.3% NaOH suprapur Type of elution: Isocratic Injection volume (µL): - Flow rate (mL/min): - Column temp: (°C): - Run time (min): -	Linearity range: - Determination coefficient (r^2^): - LOD: 0.68 µM LOQ: -	[1]
Spinach	Soluble oxalates	Column: Mightysil RP-C18 Aqua column (250 × 4.6 mm; 5 μm particle size) Guard column: - Detector (λ, nm): UV (210) Mobile phase: Tetrabutylammonium chloride (5 mM) in phosphate ammonium buffer (pH 6.8) Type of elution: Isocratic Injection volume (µL): - Flow rate (mL/min): 1 Column temp: (°C): 30 Run time (min): -	Linearity range: - Determination coefficient (r^2^): - LOD: - LOQ: -	[73]
Cereal and cereal products	Total and soluble oxalates	Column: Dionex IonPac AS4A anion exchange column (250 × 4 mm; 15 µm particle size) Guard column: - Detector (λ, nm): Amperometric Mobile phase: 2.0 g of EDTA/L of distilled water adjusted to pH 5.0 by adding 15 μL of 0.3 N NaOH Type of elution: Isocratic Injection volume (µL): - Flow rate (mL/min): - Column temp: (°C): - Run time (min): -	Linearity range: - Determination coefficient (r^2^): - LOD: - LOQ: -	[64]
Thai vegetables, cereal grains, and legume seeds	Total and soluble oxalates	Column: Bio-Rad Aminex ion exclusion HPX-87H (300 × 7.8 mm; 9 µm particle size) Guard column: - Detector (λ, nm): UV (210) Mobile phase: H_2_SO_4_ (0.0125 M) Type of elution: Isocratic Injection volume (µL): - Flow rate (mL/min): - Column temp: (°C): - Run time (min): -	Linearity range: 0.1–0.5 mg/mL Determination coefficient (r^2^): 0.997 LOD: - LOQ: 3 mg/100 g	[61]
Korean vegetables	Total and soluble oxalates	Column: Bio-Rad Aminex ion exclusion HPX-87H (300 × 7.8 mm; 9 µm particle size) Guard-column: Aminex Cation-H guard column Detector (λ, nm): UV (215) Mobile phase: H_2_SO_4_ (0.008 N) Type of elution: Isocratic Injection volume (µL): 20 Flow rate (mL/min): 0.6 Column temp: (°C): - Run time (min): -	Linearity range: 0.0168–1.3131 mg/mL Determination coefficient (r^2^): 0.9995 LOD: - LOQ: -	[40]
Taro leaves and corms and Indian vegetables	Total and soluble oxalates	Column: Bio-Rad Aminex ion exclusion HPX-87H (300 × 7.8 mm; 9 µm particle size) Guard column: Aminex Cation-H guard column Detector (λ, nm): UV–Vis (210) Mobile phase: H_2_SO_4_ (0.0125 M) filtered through a 0.45 µm membrane and degassed using vacuum Type of elution: Isocratic Injection volume (µL): 5 Flow rate (mL/min): 0.5 (0.1 prior to use and in between sample sets) Column temp: (°C): RT Run time (min): -	Linearity range: - Determination coefficient (r^2^): - LOD: 5 mg/100 g DM LOQ: -	[34]
Pakistani vegetables and beans	Total and soluble oxalates	Column: Supelco reversed-phase column (250 × 4.6 mm; 5 µm particle size) Guard column: - Detector (λ, nm): UV (210) Mobile phase: 0.25% dihydrogenate phosphate and 0.0025 M tetrabutylammonium hydrogen sulfate buffered at pH 2.0 with ortho-phosphoric acid Type of elution: Isocratic Injection volume (µL): 5 Flow rate (mL/min): 1 Column temp: (°C): - Run time (min): -	Linearity range: 1 × 10^−3^–0.04 mg/mL Determination coefficient (r^2^): 0.9773 LOD: - LOQ: -	[36]
Spinach	Total and soluble oxalates	Column: Rezex ROA ion exclusion organic acid column (300 × 7.8 mm; 8 µm particle size) Guard column: Bio-Rad cation-H guard column Detector (λ, nm): UV–Vis (210) Mobile phase: H_2_SO_4_ (25 mM) Type of elution: Isocratic Injection volume (µL): 20 Flow rate (mL/min): 0.6 Column temp: (°C): 25 Run time (min): -	Linearity range: - Determination coefficient (r^2^): - LOD: - LOQ: -	[66]
Green and black tea	Soluble oxalates	Column: Shodex IC SI-90 anion exchange column (250 × 4 mm; 9 μm particle size) filled with KanK-ASt (120 × 5 mm, 14 μm particle size) Guard column: - Detector (λ, nm): Conductivity Mobile phase: Na_2_CO_3_ (1.9 mM) and NaHCO_3_ (2.4 mM) solution Type of elution: Isocratic Injection volume (µL): 20 Flow rate (mL/min): 1.5 Column temp: (°C): 33 Run time (min): -	Linearity range: 1 × 10^−4^–0.02 mg/mL Determination coefficient (r^2^): 0.9998 LOD: 3 × 10^−5^ mg/mL LOQ: 1 × 10^−4^ mg/mL	[71]
Spinach	Total and soluble oxalates	Column: Hypersil C18 column (250 × 4.6 mm; 5 μm particle size) Guard column: - Detector (λ, nm): UV (220) Mobile phase: Aqueous solution containing 0.5% KH_2_PO_4_ and 0.5 mM tetra-*n*-butyl ammonium hydrogen sulfate (pH 2.0) degassed with an ultrasonic generator for 20 min Type of elution: Isocratic Injection volume (µL): 5 Flow rate (mL/min): 0.5 Column temp: (°C): 40 Run time (min): -	Linearity range: - Determination coefficient (r^2^): - LOD: - LOQ: -	[39]
Green juices with spinach and other vegetables and fruits	Total and soluble oxalates	Column: Rezex ROA ion exclusion organic acid column (300 × 7.8 mm; 8 µm particle size) Guard column: Bio-Rad cation-H guard column Detector (λ, nm): UV–*Vis* (210) Mobile phase: H_2_SO_4_ (25 mM) Type of elution: Isocratic Injection volume (µL): 20 Flow rate (mL/min): 0.6 Column temp: (°C): - Run time (min): -	Linearity range: 0.01–0.25 mg/mL Determination coefficient (r^2^): - LOD: - LOQ: -	[67]
Spinach	Total oxalates	Column: Rezex ROA ion exclusion organic acid column (300 × 7.8 mm; 8 µm particle size) Guard column: Bio-Rad cation-H guard column Detector (λ, nm): UV–*Vis* (210) Mobile phase: H_2_SO_4_ (25 mM) Type of elution: Isocratic Injection volume (µL): 20 Flow rate (mL/min): 0.6 Column temp: (°C): 25 Run time (min): -	Linearity range: 0.01–0.25 mg/mL Determination coefficient (r^2^): - LOD: - LOQ: -	[65]
Alcoholic and non-alcoholic beverages	Soluble oxalates	Column: Dionex IonPac AS4A anion exchange column (250 × 4 mm; 15 µm particle size) Guard column: - Detector (λ, nm): Amperometric Mobile phase: Aqueous EDTA solution (2.0 g/L) adjusted to pH 5.0 with 0.3 M NaOH Type of elution: Isocratic Injection volume (µL): - Flow rate (mL/min): 0.6 Column temp: (°C): - Run time (min): -	Linearity range: - Determination coefficient (r^2^): - LOD: - LOQ: -	[72]
Vegetables and fruits	Total oxalates	Column: Agilent Poroshell-C18 (250 × 4.6 mm; 2.7 μm particle size) Guard column: - Detector (λ, nm): UV (210) Mobile phase: 50 mM KH_2_PO_4_, H_3_PO_4_ (pH 2.8) Type of elution: Isocratic Injection volume (µL): 5 Flow rate (mL/min): 1 Column temp: (°C): 50 Run time (min): 25	Linearity range: 0.1–1.0 mg/mL Determination coefficient (r^2^): - LOD: - LOQ: -	[58]
		Column: Agilent Poroshell-C18 (100 × 2.1 mm; 2.7 μm particle size) Guard column: - Detector (λ, nm): UV (210) Mobile phase: 50 mM KH_2_PO_4_, H_3_PO_4_ (pH 2.8) Type of elution: Isocratic Injection volume (µL): 5 Flow rate (mL/min): 0.6 Column temp: (°C): 20 Run time (min): 25		
Taro corms	Total and soluble oxalates	Column: Rezex ROA ion exclusion organic acid column (300 × 7.8 mm; 8 µm particle size) Guard column: Cation H-guard column Detector (λ, nm): DAD (210) Mobile phase: 60% H_2_SO_4_ (0.005 N) and 40% CH_3_CN Type of elution: Isocratic Injection volume (µL): 5 Flow rate (mL/min): 0.5 Column temp: (°C): 50 Run time (min): 20	Linearity range: 6.25 × 10^−3^–0.2 mg/mL Determination coefficient (r^2^): 0.999 LOD: - LOQ: -	[68]
Mexican vegetables	Total and soluble oxalates	Column: Sphereclone ODS-column (250 × 4.6 mm; 5 µm particle size) Guard column: - Detector (λ, nm): UV–*Vis* (215) Mobile phase: H_2_SO_4_ (1.8 μM) in distilled water (pH 2.6) Type of elution: Isocratic Injection volume (µL): 20 Flow rate (mL/min): 0.4 Column temp: (°C): - Run time (min): -	Linearity range: - Determination coefficient (r^2^): - LOD: - LOQ: -	[56]
Beans, lentils, sweet potato, and others	Total and soluble oxalates	Column: Dionex IonPac AS4A anion exchange column (250 × 4 mm; 15 µm particle size) Guard column: - Detector (λ, nm): Amperometric Mobile phase: Aqueous EDTA solution (2.0 g/L) adjusted to pH 5.0 with 0.3 M NaOH Type of elution: Isocratic Injection volume (µL): - Flow rate (mL/min): - Column temp: (°C): - Run time (min): -	Linearity range: - Determination coefficient (r^2^): - LOD: - LOQ: -	[63]

DM—dry matter; LOD—limit of detection; LOQ—limit of quantification; temp—temperature; “-“—information not available.

**Table 4:** Spectrophotometric conditions for the analysis of oxalates in foods.

Matrix	Analytes	Reaction ^(a)^	Spectrophotometric Conditions	Standard Solution	Validation Data	References
Fruits, beverages, and vegetables	Soluble oxalates	Oxidation of oxalate by oxygen in the presence of oxalate oxidase, forming hydrogen peroxide, which will form a monoperoxo complex: (COOH)_2_ + O_2_ → 2CO_2_ + H_2_O_2_ TiO(tpypH_4_)^4+^ + H_2_O_2_ → **TiO_2_(tpypH_4_)^4+^** + H_2_O.	Temperature of reaction (°C): 75 pH of reaction: 3 Time of reaction (min): - Oxalate effect: - Wavelength (nm): 450	Sodium oxalate	Linearity range (μg/mL): 0.067–33.5 Determination coefficient (r^2^): 0.998 LOD (μg/mL): - LOQ (μg/mL): -	[77]
Spinach	Total oxalates	Oxidation of **rhodamine B** by potassium dichromate in sulfuric acid.	Temperature of reaction (°C): 90 pH of reaction: - Time of reaction (min): 8 Oxalate effect: Catalyst Wavelength (nm): 555	Oxalic acid	Linearity range (μg/mL): 0.06–40 Determination coefficient (r^2^): - LOD (μg/mL): 0.02 LOQ (μg/mL): -	[38]
Vegetables	Soluble oxalates	Oxidation of **bromophenol blue** by potassium dichromate in dilute sulfuric acid media.	Temperature of reaction (°C): 60 pH of reaction: - Time of reaction (min): 10 Oxalate effect: Catalyst Wavelength (nm): 600	Oxalic acid	Linearity range (μg/mL): 0.1–8.0 Determination coefficient (r^2^): 0.998 LOD (μg/mL): 0.04 LOQ (μg/mL): -	[79]
Spinach	Total oxalates	Oxidation of **brilliant cresyl blue** by potassium dichromate in acidic media.	Temperature of reaction (°C): 80 pH of reaction: - Time of reaction (min): - Oxalate effect: Catalyst Wavelength (nm): 625	Oxalic acid	Linearity range (μg/mL): 0.020–4.70 Determination coefficient (r^2^): 0.996 LOD (μg/mL): 0.005 LOQ (μg/mL): -	[37]
Spinach	Soluble oxalates	Oxidation of **safranine** by potassium dichromate in dilute sulfuric acid media.	Temperature of reaction (°C): 60 pH of reaction: - Time of reaction (min): - Oxalate effect: Catalyst Wavelength (nm): 530	Oxalic acid	Linearity range (μg/mL): 0.10–10.0 Determination coefficient (r^2^): 0.998 LOD (μg/mL): 0.08 LOQ (μg/mL): -	[31]
Spinach	Soluble oxalates	Oxidation of Mn(II) to MnO_4_^−^ by potassium periodate: MnSO_4_ + KIO_4_ → **MnO_4_^−^** + 2IO_3_^−^	Temperature of reaction (°C): 35 pH of reaction: - Time of reaction (min): 18 Oxalate effect: Catalyst Wavelength (nm): 525	Sodium oxalate	Linearity range (μg/mL): 0.05–1.25 and 0.05–1.75 Determination coefficient (r^2^): 0.998 (for the range of 0.05–1.25 μg/mL) LOD (μg/mL): 0.027 and 0.005 LOQ (μg/mL): -	[80]
Spinach and mushrooms	Total oxalates	Oxidation of **pyrocathecol violet** with potassium dichromate in acidic media.	Temperature of reaction (°C): 30 pH of reaction: - Time of reaction (min): - Oxalate effect: Catalyst Wavelength (nm): 450	Oxalic acid	Linearity range (μg/mL): 0.08–1.30 Determination coefficient (r^2^): 0.9993 LOD (μg/mL): 0.07 LOQ (μg/mL): -	[74]
Spinach	Soluble oxalates	Oxidation of **Victoria blue B** by potassium dichromate in dilute sulfuric acid media.	Temperature of reaction (°C): 60 pH of reaction: - Time of reaction (min): 9 Oxalate effect: Catalyst Wavelength (nm): 610	Oxalic acid	Linearity range (μg/mL): 0.06–9.0 Determination coefficient (r^2^): ≥ 0.996 LOD (μg/mL): 0.12 LOQ (μg/mL): -	[81]
Spinach and mushrooms	Soluble oxalates	Oxidation of iodide by bromate in acidic media catalyzed by iron(II) in the presence of oxalate ion as activator: BrO_3−_ + 9I^−^ + 6H^+^ → **3I_3_**_−_ + 3H_2_O + Br^−^.	Temperature of reaction (°C): 20 pH of reaction: 5 Time of reaction (min): - Oxalate effect: Activator Wavelength (nm): 352	Sodium oxalate	Linearity range (μg/mL): 0.10–7.0 Determination coefficient (r^2^): 0.998 LOD (μg/mL): 0.08 LOQ (μg/mL): -	[54]
Spinach, beetroot, and mushrooms	Soluble oxalates	Oxidation of **Victoria blue 4R** by dichromate in acidic media.	Temperature of reaction (°C): 25 pH of reaction: 4 Time of reaction (min): - Oxalate effect: Catalyst Wavelength (nm): 615	Oxalic acid	Linearity range (μg/mL): 2.0–180 Determination coefficient (r^2^): 0.995 LOD (μg/mL): 0.7 LOQ (μg/mL): -	[82]
Tap water	Soluble oxalates	Reduction of **copper(II) complex** to copper (I) by oxalate ion.	Temperature of reaction (°C): RT pH of reaction: - Time of reaction (min): 10 Oxalate effect: - Wavelength (nm): 533	Sodium oxalate	Linearity range (μg/mL): 0.1–2.0 Determination coefficient (r^2^): - LOD (μg/mL): - LOQ (μg/mL): -	[75]
Spinach and mushrooms	Soluble oxalates	Oxidation of **crystal violet** by potassium dichromate in sulfuric acid media.	Temperature of reaction (°C): 20 pH of reaction: - Time of reaction (min): - Oxalate effect: Catalyst Wavelength (nm): 630	Sodium oxalate	Linearity range (μg/mL): 0.2–1.8 and 1.8–5.5 Determination coefficient (r^2^): 0.993 and 0.994 LOD (μg/mL): 0.05 LOQ (μg/mL): -	[78]
Vegetables	Total and soluble oxalates	Ion association of stable anionic complex, which is produced by the reaction of oxalate with Mo(VI) and with Toluidine blue (TBH^2+^): (MoO_4_)^2−^ + Ox^2−^ ↔ [MoO_3_(Ox)]^2−^ + H_2_O [MoO_3_(Ox)]^2−^ + TBH^2+^ ↔ **TBH^2+^[MoO_3_(Ox)]** or [MoO_3_(Ox)]^2−^ + 2TB^+^ ↔ **(TB)_2_[MoO_3_(Ox)]**.	Temperature of reaction (°C): - pH of reaction: 6 Time of reaction (min): - Oxalate effect: - Wavelength (nm): 627	Sodium oxalate	Linearity range (μg/mL): 0.0012- 0.012 and 0.012–0.240 Determination coefficient (r^2^): 0.9974 and 0.9915 LOD (μg/mL): 0.00036 LOQ (μg/mL): 0.0012	[30]
Vegetables and aromatic plants	Soluble oxalates	Interaction of oxalates with a sensor material of Si-Ti/ECR, silica–titania xerogel with eriochrome cyanine R.	Temperature of contact (°C): - pH of contact: 3 Time of contact (min): 15 Oxalate effect: - Wavelength (nm): 570	Oxalic acid	Linearity range (μg/mL): 35–900 Determination coefficient (r^2^): 0.9982 LOD (μg/mL): 10.5 LOQ (μg/mL): 35	[41]
Spinach and mushrooms	Soluble oxalates	Reaction of Reactive blue 4-Cu^2+^ with oxalate: **RB4-Cu^2+^** + (C_2_O_4_)^2−^ → CuC_2_O_4_ + **RB4**.	Temperature of reaction (°C): - pH of reaction: 5–7 Time of reaction (min): - Oxalate effect: - Wavelength (nm): 607	Potassium oxalate	Linearity range (μg/mL): 0.29–8.21 Determination coefficient (r^2^): 0.9983 LOD (μg/mL): 0.10 LOQ (μg/mL): 0.34	[76]
Vegetables and fruits	Total oxalates	Reduction of **hexavalent chromium** by oxalic acid in presence of Mn(II) as a catalyst.	Temperature of reaction (°C): 25 pH of reaction: 3 Time of reaction (min): 60 Oxalate effect: - Wavelength (nm): 350	Potassium oxalate	Linearity range (μg/mL): 1.660–332.4 Determination coefficient (r^2^): 0.997 LOD (μg/mL): 0.20 LOQ (μg/mL): 0.66	[58]

^a^ Chemical compounds in **bold** are the substances whose absorbance is measured in each reaction. LOD—limit of detection; LOQ—limit of quantification. “-“—information not available.

**Table 5:** Occurrence of oxalate (mg/100 g of fresh weight) in foods.

Food	n	Type of Sample	Total Oxalates (mg/100 g FW)	Soluble Oxalates (mg/100 g FW)	Insoluble Oxalates (mg/100 g FW)	References
Amaranth *Amaranthus mangostamus*	-	Raw	1510.8	835.1	675.7	[40]
Apple *Malus domestica*	1	Granny Smith, raw	3.5	1.8	1.7	[1]
Apple *Malus sylvestris*	26	Cox Kent, raw	-	0.4	0.6	
	28		1	-		
Apricot *Prunus armeniaca*	2	Raw	6.8	1.9	4.9	
Artichoke *Cynara scolymus*	1	Boiled	6.8	6.8	0	
Arugula *Eruca vesicaria*	10	Raw	-	ND	-	[59]
Asparagus *Asparagus officinalis*	3	Boiled	2.6	0.9	1.7	[1]
Asparagus chicory *Cichorium intybus*	10	Raw	-	1	-	[59]
Aubergine *Solanum melogena* (or eggplant)	3	Green, long, and raw; boiled	55; 38	45; 19	10; 19	[61]
	-	Raw	54.4	53.7	0.7	[40]
	1	Boiled	12.8	4.8	8	[1]
	2	Raw	16.2	15.7	0.5	
Avocado *Persea gratissima*	2	Raw	1.3	1.3	0	
Bamboo shoot *Bambusa* spp.	3	Cultivated and raw; boiled	222; 93	163; 51	60; 42	[61]
	3	Pickled and raw; boiled	71; 51	23; 10	47; 41	
Banana *Musa paradisiaca*	7	Raw	6.8	0.7	6.1	[1]
Bean *Phaseolus vulgaris*	2	Preserved white	54.2	1.9	52.3	
	2	White, seeds, dry	547.9	38.8	509.1	[63]
	3	White, raw; boiled	158; 47	52; 12	106; 34	[36]
	3	Red, raw; boiled	113; 72	37; 22	76; 50	
	1	Quail, seeds, dry	176.7	16.9	159.8	[63]
	3	Red kidney, seeds, and raw; boiled	91; 32	26; 10	65; 27	[61]
	1	Kidney	-	32	-	[79]
	1	Red kidney	13.9	1.5	12.4	[1]
	1	Red kidney, seeds, dry	74.6	4.8	69.8	[63]
	1	Green, raw	65.2	8.4	56.8	
Beetroot *Beta vulgaris*	3	Raw; boiled	67; 52	45; 38	22; 15	[36]
	5	Raw	-	1431	-	[82]
	2	Boiled	36.9	16.3	20.6	[1]
	6	Raw	-	74,9	-	[59]
	-	Raw; boiled	45.6; 76.0	38.6; 72.3	7; 3.7	[32]
Bellflower root *Platycodon grandiflorum*	-	Raw	ND	ND	-	[40]
Bilberry *Vaccinium myrtillus*	3	Raw	1.5	0.1	1.4	[1]
Blackcurrent *Ribes nigrum*	1	Raw	19	3	16	
Bramble (blackberry) *Rubus fruticosus*	4	Raw	29.2	0.9	28.3	
Broccoli *Brassica oleracea*	-	Raw	ND	ND	-	[40]
	2	Boiled	1.4	1.1	0.3	[1]
	5	Raw	-	0,5	-	[59]
	-	Raw; boiled	16.1; 10.1	11.6; 6.6	4.5; 3.5	[32]
Broccoli raab *Brassica rapa*	10	Raw	-	0,1	-	[59]
Brussel sprout *Brassica oleracea*	3	Boiled	1.2	0.8	0.4	[1]
Burdock *Arctium lappa*	1	Raw	64.8	62.7	2.1	[40]
Cabbage *Brassica oleracea*	-	Raw	ND	ND	-	
	3	Raw; boiled	7; 5	ND; 4	7; <DL	[61]
	3	Raw	-	ND	-	[59]
Carrot *Daucus carota*	3	Raw; boiled	29; 12	24; 7	5; 5	[61]
	3	Raw; boiled	49; 26	28; 14	21; 12	[36]
	-	Raw	16.4	16.2	0.2	[40]
	24	Raw	17.8	9.0	8.8	[1]
	1	Boiled	4.9	2.3	2.6	
	7	Raw	-	12	-	[59]
	-	Raw; boiled	35.6; 32.3	22.6; 19.3	13.0; 13.0	[32]
Cauliflower *Brassica oleracea*	1	Raw	-	0.3	0.1	[1]
	2		0.4	-		
	4	Raw	-	ND	-	[59]
	3	Raw; boiled	27; 8	ND; ND	27; 6	[61]
Celery *Apium graveolens*	-	Raw	23.2	ND	23.2	[40]
	1	Canned	6.7	3.5	3.2	[1]
	6	Raw	-	0,5	-	[59]
Chanterelles *Cantharellus cibarius*	2	Canned	0.5	0.5	0	[1]
Cherry *Prunus avium*	5	Sweet, raw	2.4	1.3	1.1	
Chickpeas *Cicer arietinum*	2	Seeds, dry	14.3	13.7	0.6	[63]
Chinese convolvulus *Lpomoea reptans*	3	Raw; boiled	156; 135	21; 7	135; 128	[61]
Chinese kale *Brassica oleracea*	3	Raw; boiled	23; 7	ND; ND	22; 7	
Chinese cucumber *Momordica charantia*	3	Raw; boiled	71; 56	57; 22	14; 34	
Coriander *Coriandrum sativum*	-	Dried	40.6	ND	40.6	[33]
	4	Raw, air dried	40.5	ND	40.5	[34]
Cowpea *Vigna unguiculata*	3	Seeds and raw; boiled	ND; 5	ND; ND	ND; 5	[61]
Cress *Nasturtium officinale*	1	-	ND	ND	-	[1]
Crown daisy *Chrysanthemum coronarium*	-	Raw	96.0	58.8	37.2	[40]
Cucumber *Cucumis sativus*	-	Raw	ND	ND	-	
	1	Raw	0.4	0.3	0.1	[1]
Elderberry *Sambucus nigra*	4	Black, raw	72.1	7.1	65	
Endive *Cichorium endivia*	5	Raw	-	0.2	-	[59]
Fava beans *Vicia faba*	1	Seeds, raw	1.3	0.9	0.4	[63]
Fennel *Foeniculum vulgare*	2	Boiled	5.3	3.3	2	[1]
	1	Raw	19.7	17.2	2.5	
	11	Raw	-	12.4	-	[59]
Fig *Ficus carica*	3	Raw	20.5	3.3	17.2	[1]
	2	Dried	-	5.5	89.6	
	1		95.1	-		
Garlic *Allium sativum*	-	Raw	ND	ND	-	[40]
	4	Raw	-	ND	-	[59]
Green amaranth *Amaranthus viridis*	-	Leaves, dried	1939.9	901.7	1038.1	[33]
	4	Raw, air dried	1940.8	902.3	1038.7	[34]
Green peas *Pisum sativum*	2	Seeds, dry	3.3	2.7	0.6	[63]
Green onion *Allium cepa*	3	Raw	-	ND	-	[59]
Green pepper *Capsicum annuum*	-	Raw	31.0	27.5	3.5	[40]
Gooseberry *Grossularia uva crispa*	1	Red	21.6	3.2	18.4	[1]
	1	Green, raw	27	3.1	23.9	
Granadilla *Passiflora edulis*	1	Raw	1	0.6	0.4	
Grape *Vitis vinifera*	9	Green, raw	1.7	0.6	1.1	
Huauzontle *Chenopodium Nuttalliae*	3	Raw; boiled	162.0; 97.7	135.0; 97.7	27.1; 0	[56]
Ivy gourd *Coccinia grandis*	3	Raw; boiled	36; 24	10; 5	29; 19	[61]
Kale *Brassica oleracea*	-	Raw	ND	ND	-	[40]
Kiwi fruit *Actinida chinesis*	-	-	-	4.50	-	[77]
	6	Raw	23	2.4	20.6	[1]
Kohlrabi *Brassica oleracea*	1	Boiled	0.7	0.7	0	
	3	Raw	-	ND	-	[59]
Leaf chicory *Cichorium intybus*	-	Raw	47.6	42.3	5.3	[40]
	8	Raw	-	ND	-	[59]
Leek *Allium porrum*	2	Raw	17.0	9.4	7.6	[1]
Leek *Allium tuberosum*	-	Raw	48.6	45.1	3.5	[40]
Lemon *Citrus medica*	2	Raw	3.1	0.5	2.6	[1]
Lentil *Lens culinaris*	2	Brown, seeds, dry	24.0	14.3	9.7	[63]
	2	Red, seeds, dry	13.8	7.8	6	
Lentil *Lens esculenta*	1	Dried	13.3	1.9	11.4	[1]
Lettuce *Lactuca sativa*	-	Raw	40	ND	40	[40]
	2	Raw	0.3	0.3	0	[1]
	16	Raw	-	ND	-	[59]
Lettuce *Valeriana locusta*	1	Raw	1.3	0.9	0.4	[1]
Licorice *Glycyrrhiza glabra*	2	Root	3569.3	165	3404.3	[63]
Lime *Citrus auranttifolia*	1	Raw	7.5	0.5	7	[1]
Lotus root *Nelumbo nucifera*	-	Raw	ND	ND	-	[40]
Mandarin *Citrus nobilis*	2	Raw	8.5	0.3	8.2	[1]
Mango *Magnifera indica*	4	Raw	-	0.5	1.1	
	5		1.6	-		
Mirabelle *Prunus domestica syriaca*	4	Raw	8.1	0.2	7.9	
Mung bean *Vigna radiata*	3	Seeds and raw; boiled	24; 5	12; ND	12; 4	[61]
Mushroom *Agaricus bisporus*	5	Raw	-	320	-	[78]
	1	Raw	-	41	-	[79]
	5	Raw	-	36	-	[82]
	1	Raw		-	-	[74]
	5	Raw	-	326	-	[54]
	3	Canned	0.7	0.4	0.3	[1]
	1	Boiled	0.5	0.1	0.4	
	-	Raw	-	482	-	[76]
Muskmelon *Cucumis melo*	6	Raw	-	0.9	0.1	[1]
	7		1	-		
New Zealand Spinach *Tetragonia tetragonioides*	-	Raw; boiled	1764.7; 1322.6	364.6; 129.3	1400.1; 1193.3	[32]
Okra *Abdelmoschus esculentus*	1	Raw	317.2	56.3	260.9	[63]
Olive *Olea europaea*	2	Green, canned	45.7	1.2	44.5	[1]
	3	Black, canned	13.9	1.6	12.3	
Onion *Allium cepa*	-	Raw	ND	ND	-	[40]
	2	Raw	1.7	1.6	0.1	[1]
	6	Raw	-	ND	-	[59]
Onion stalks *Allium cepa*	-	Dried	29.1	ND	29.1	[33]
	4	Raw, air dried	29.3	ND	29.3	[34]
Orange *Citrus sinsensis*	1	Raw	1.8	0.2	1.6	[1]
Oval kumquat *Fortunella margarita*	1	Raw	3.5	0.8	2.7	
Papaloquelite *Porophyllum ruderale*	3	Raw	15.8	15.8	ND	[56]
Papaya *Carica papaya*	3	Raw; boiled	5; 11	ND; ND	ND; 8	[61]
	3	Raw	1.3	0.5	0.8	[1]
Parsley	3	Raw	-	782	-	[41]
Parsley *Petroselinum sativum*	1	-	-	76	60	[1]
	2		136	-		
Parsley *Petroselinum crispum*	-	Raw	270.7	72.0	198.8	[40]
	8	Raw	-	0.5	-	[59]
Pea *Pisum sativum*	1	Canned	6.2	6.2	0	[1]
	1	Boiled	0.2	0.2	0	
	2	Green, dried	ND	ND	-	
Peach *Prunus persica*	3	Raw	2.5	0.2	2.3	
Pear *Pyrus communis*	10	Raw	-	0.9	1.8	
	11		2.7	-		
	2	Peeled	-	3.8	-	
	3		3.7			
Persimmon *Diospyros kaki*	-	-	-	2.61	-	[77]
Pineapple *Ananas comosus*	2	Preserved without sugar	-	0.9	4	[1]
	3		4.9	-		
Plum *Prunus domestica*	8	Raw	1.7	0.5	1.2	
Potato *Solanum tuberosum*	3	Boiled	24.3	12.8	11.5	
	1	Baked	13.0	11.7	1.3	
	1	Deep fried	26.9	17.0	9.9	
	1	Chips	47.0	45.8	1.2	
	2	Raw	17.1	13.0	4.1	
	3	Raw	-	0,4	-	[59]
Pumpkin *Cucurbita pepo*	-	Raw	ND	ND	-	[40]
	1	Raw	ND	ND	-	[1]
Purple amaranth *Amaranthus cruentus*	-	Leaves, dried	1355.3	594.9	760.4	[33]
	4	Raw, air dried	1353.7	594.2	759.5	[34]
Quelite *Chenopodium album*	3	Raw; boiled	110.0; 59.8	72.4; 31.0	37.6; 28.9	[56]
Radicchio *Cichorium intybus*	3	Raw	-	ND	-	[59]
Radish *Raphanus sativus*	-	White	-	1.46	-	[77]
	1	Raw, red	1.7	1.4	0.3	[1]
	1	Raw, white	ND	ND	-	
Radish leaves	-	Dried	12.3	ND	12.3	[33]
	4	Raw, air dried	12.3	ND	12.3	[34]
	6	Raw	-	ND	-	[59]
Radish roots	-	Raw	ND	ND	-	[40]
	6	Raw	-	ND	-	[59]
Raspberry *Rubus idaeus*	4	Raw	18.9	3.4	-	[1]
Redcurrant *Ribes rubrum*	4	Raw	19.8	4.9	-	
Rhubarb *Rheum rhabarbarum*	1	Raw	-	380	855	
	2		1235	-		
Rhubarb petioles	4	Raw	1080	-	-	[55]
Rhubarb stalks	-	Raw; boiled	986.7; 756.3	287.3; 80.7	699.4; 675.6	[32]
Romerito *Suaeda torreyana*	3	Raw; boiled	94.2; 24.0	67.0; 11.2	27.2; 12.8	[56]
Sauerkraut *Brassica oleracea*	1	Raw	7.1	7.1	0	[1]
Savoy cabbage *Brassica oleracea*	1	Boiled	3.5	1.3	2.2	
	2	Raw	-	0.1	-	[59]
Sorrel *Rumex acetosa*	3	Raw	-	1079	-	[41]
Soybean *Glycine max*	3	Seeds, raw	204	58	145	[61]
	3	Raw; boiled	497; 224	155; 64	343; 162	[36]
	1	Flakes	218.4	32.6	185.8	[63]
	1	Flour	124	29.9	94,1	
	2	Seeds, dry	276.8	37.9	238.9	
Soybean sprout	1	Raw	26.5	12.7	13.8	[40]
Spinach *Spinacia oleracea*	-	Raw	2350	2000	350	[39]
	-	Raw	460	-	-	[38]
	10	Raw	390	-	-	[37]
	2	Raw	-	731	-	[80]
	5	Raw	-	762	-	[54]
	3	Raw	-	2.61	-	[81]
	5	Raw	-	415	-	[82]
	2	Raw		-	-	[74]
	3	Raw	-		-	[79]
	-	Raw	-	421	-	[31]
	5	Raw	-	1330	-	[78]
	-	-	-	515	-	[77]
	182	Frozen, cultivated in winter	-	1092.9	-	[73]
		Frozen, cultivated in spring	-	890.3	-	
		Frozen, cultivated in summer	-	752.5	-	
		Frozen, cultivated in fall	-	614.9	-	
	3	Raw; boiled	978; 477	543; 184	435; 293	[36]
	2	Boiled	364	101	263	[1]
	1	Boiled with cream	412	123	289	
	-	Raw	1272.2	1176.1	96.0	[40]
	12	Raw	-	542.6	-	[59]
	-	Raw; boiled	329.6; 154.8	266.2; 90.9	63.4; 63.9	[32]
	1	Raw	1370	90	1280	[35]
	-	Dried	768.4	727.1	41.3	[33]
	4	Raw, air dried	691.7	643.5	48.2	[34]
	-	Raw	-	631	-	[76]
Spring onion *Allium fistulosum*	-	Raw	33.3	28.2	5.1	[40]
Star fruit *Averrhoa carambola*	4	Raw	295.4	138.9	156.5	[1]
	-	Raw	160	90	70	[35]
Strawberry *Fragaria*	8	Raw	2.9	0.9	2	[1]
Sultana *Vitis vinifera*	1	Dried	8.5	3.2	5.3	
Swamp morning glory *Ipomoea aquatica*	3	White stems and raw; boiled	79; 56	58; 40	21; 16	[61]
	3	Red stems and raw; boiled	94; 59	61; 23	33; 36	
Sweet potato *Ipomea batatas*	2	Raw	495.6	76.7	418.9	[63]
	3	Raw	-	ND	-	[59]
Swiss chard *Beta vulgaris*	6	Raw	874	327	547	[1]
	-	Raw	1458.1	1082.7	375.4	[40]
	12	Raw	-	207,7	-	[59]
Swiss chard stems	-	Raw; boiled	127.4; 148.4	24.0; 19.4	103.4; 129.0	[32]
Swiss chard leaves	-	Raw; boiled	525.5; 291.1	252.3; 117.7	273.2; 173.4	[32]
Taro leaves *Colocasia esculenta*	4	Maori cultivar, raw	443.7	204.1	239.5	[34]
	4	Maori cultivar, baked	721.9	367.2	354.7	
	4	Maori cultivar, baked with milk	397.5	173.3	224.2	
	4	Japanese cultivar, raw	424.7	267.0	157.7	
	4	Japanese cultivar, baked	533.9	352.6	181.3	
	4	Japanese cultivar, baked with milk	300.2	144.6	155.6	
Tassel hyacinth *Leopoldia comosa*	3	Raw	-	ND	-	[59]
Tamarillo *Tamarindus indica*	1	Raw	19.9	3.7	16.2	[1]
Tomato *Lycopersicum esculentum*	3	Raw	11	7	4	[61]
	3	Raw	8.5	3.6	4.9	[1]
	1	Canned, peeled	12.7	3.1	9.6	
Verdolaga *Portulaca oleracea*	3	Raw; boiled	91.5; 119.8	62.7; 50.1	28.7; 69.7	[56]
Viper’s grass *Scorzonera hispanica*	1	Canned	9.1	6.5	2.6	[1]
Water dropwort *Oenanthe javanica*	-	Raw	93.0	64.7	28.3	[40]
Watermelon *Citrullus lanatus*	1	Raw	0.3	0.3	0	[1]
Winged bean *Psophocarpus tetragonolobus*	3	Pods and raw; boiled	7; 5	ND; ND	4; 5	[61]
Yard long bean *Vigna sesquipedalis*	3	Green and raw; boiled	38; 29	9; 3	29; 23	
Yellow plum *Prunus domestica* ssp *syriaca*	2	Raw	1.4	0.4	1	[1]

ND—not detected; <DL—below limit of detection; n—number of samples; FW—fresh weight.

## Data Availability

Data is contained within the article.
